# Advancing Drug Delivery Paradigms: Polyvinyl Pyrolidone (PVP)-Based Amorphous Solid Dispersion for Enhanced Physicochemical Properties and Therapeutic Efficacy

**DOI:** 10.3390/polym16020286

**Published:** 2024-01-20

**Authors:** Agus Rusdin, Amirah Mohd Gazzali, Nur Ain Thomas, Sandra Megantara, Diah Lia Aulifa, Arif Budiman, Muchtaridi Muchtaridi

**Affiliations:** 1 Department of Pharmaceutical Analysis and Medicinal Chemistry, Faculty of Pharmacy, Universitas Padjadjadjaran, Jl. Raya Bandung-Sumedang Km-21, Bandung 45363, Indonesia; agusrusdin@gmail.com (A.R.); s.megantara@unpad.ac.id (S.M.); diah.lia@unpad.ac.id (D.L.A.); 2 Department of Pharmaceutics and Pharmaceutical Technology, Faculty of Pharmacy, Universitas Padjadjadjaran, Jl. Raya Bandung-Sumedang Km-21, Bandung 45363, Indonesia; arif.budiman@unpad.ac.id; 3 Departement Pharmaceutical Technology, School of Pharmaceutical Sciences, Universiti Sains Malaysia, P.Penang, Penang 11800, Malaysia; amirahmg@usm.my; 4 Department of Pharmacy, Faculty of Sport and Health, Universitas Negeri Gorontalo, Jl. Jenderal Sudirman No. 6, Gorontalo 96128, Indonesia; nurain.thomas@gmail.com; 5 Research Collaboration Centre for Theranostic Radiopharmaceuticals, National Research and Innovation Agency (BRIN), Jakarta Pusat 10340, Indonesia

**Keywords:** amorphous solid dispersion, PVP-based, drug delivery, solubility

## Abstract

Background: The current challenge in drug development lies in addressing the physicochemical issues that lead to low drug effectiveness. Solubility, a crucial physicochemical parameter, greatly influences various biopharmaceutical aspects of a drug, including dissolution rate, absorption, and bioavailability. Amorphous solid dispersion (ASD) has emerged as a widely explored approach to enhance drug solubility. Objective: The objective of this review is to discuss and summarize the development of polyvinylpyrrolidone (PVP)-based amorphous solid dispersion in improving the physicochemical properties of drugs, with a focus on the use of PVP as a novel approach. Methodology: This review was conducted by examining relevant journals obtained from databases such as Scopus, PubMed, and Google Scholar, since 2018. The inclusion and exclusion criteria were applied to select suitable articles. Results: This study demonstrated the versatility and efficacy of PVP in enhancing the solubility and bioavailability of poorly soluble drugs. Diverse preparation methods, including solvent evaporation, melt quenching, electrospinning, coprecipitation, and ball milling are discussed for the production of ASDs with tailored characteristics. Conclusion: PVP-based ASDs could offer significant advantages in the formulation strategies, stability, and performance of poorly soluble drugs to enhance their overall bioavailability. The diverse methodologies and findings presented in this review will pave the way for further advancements in the development of effective and tailored amorphous solid dispersions.

## 1. Introduction

The characteristics of drugs, specifically their physicochemical properties, play a crucial role in determining how drugs behave in the body. These properties have a significant impact on a drug’s pharmacokinetics (how the body processes the drug), bioavailability (how much of the drug reaches its target site), and overall performance. Solubility and permeability, which are the key physicochemical properties, are also used to categorize drugs within the biopharmaceutical classification system (BCS). Additionally, the stability of a drug, which refers to its ability to remain unchanged over time, is an important factor that affects both its effectiveness and potential toxicity [1,2,3,4].

The significance of a drug’s physicochemical properties cannot be overstated when it comes to the effectiveness of a treatment. The challenges associated with understanding and optimizing these properties have become a primary focus for researchers seeking to develop new drugs.

At present, researchers have employed diverse methods, both chemical and physical, to tackle physicochemical challenges and enhance drug efficacy. Among these techniques, amorphous solid dispersion stands out as a promising approach for modifying drugs and addressing issues related to their solubility, stability, and overall effectiveness [5,6,7,8,9].

The amorphization process is widely used to enhance the solubility of a compound with low water solubility by taking advantage of its lower Gibbs free energy compared to its crystalline form. However, amorphous substances are inherently thermodynamically unstable and tend to recrystallize easily when stored or when dispersed in water. Therefore, additional measures, such as amorphous solid dispersion, are necessary to stabilize these amorphous substances [10,11].

Amorphous solid dispersion (ASD) represents a formulation approach employed in stabilizing amorphous active ingredients. In this approach, the drug is dispersed at a molecular level within a polymer matrix, maintaining a non-crystalline state [12]. This molecular dispersion results in enhanced solubility and improved dissolution rate [13]. The polymers commonly used in the preparation of solid dispersions have demonstrated the ability to prevent drug recrystallization [14]. The presence of strong intermolecular interactions, such as hydrogen bonds, between the drug molecules and polymer chains in solid dispersion systems plays a crucial role in reducing the drug’s mobility and inhibiting recrystallization during storage and following redispersion in a solvent, particularly water [7,15,16].

Earlier studies have provided evidence showing that the technique of solid dispersion amorphous modification effectively enhanced the cytotoxic activity of the RA-XII cyclopeptide compound against RKO human colorectal cancer, both in vitro and in vivo. This increase in activity can be attributed to the improved solubility and permeability of the RA-XII cyclopeptide, which experienced a significant increase of 17.54 fold and 10.35 fold, respectively. Additionally, other research has indicated that ASD can elevate the bioavailability of gefitinib by 9.14 fold and enhance its anti-cancer activity on skin cancer cells (A431 skin carcinoma) [17].

ASD often utilizes synthetic, semi-synthetic, and natural polymers. Among these polymers, polyvinylpyrrolidone (PVP) is often employed as a polymer in ASD due to its ability to form a stable amorphous matrix with investigated drugs [18]. This can help prevent the drug from crystallizing, maintaining it in a more soluble amorphous state [19,20]. The amorphous form of a drug often exhibits higher solubility than its crystalline counterpart, which can lead to improved dissolution and absorption in the gastrointestinal tract [21]. PVP acts as a carrier or matrix for the drug in the ASD, helping to maintain the drug in a stable and amorphous form. This can be particularly beneficial for drugs with low aqueous solubility, as it enhances their dissolution rate and, consequently, their bioavailability [22,23,24,25].

To date, there is a notable gap in the literature whereby no systematic and comprehensive review is available that addressed the role of PVP in ASDs. Thus, this review aims to fill this crucial void by providing a thorough synthesis and analysis of the diverse applications of PVP in the realm of ASDs. By offering a consolidated overview, this article will not only address this knowledge gap but also serves as a catalyst for scientists and researchers to delve into the intricacies of utilizing PVP for the development of drugs characterized by superior physicochemical attributes and heightened efficacy. This unique contribution positions the review as an instrumental resource for advancing the understanding and innovation in the field, paving the way for the creation of pharmaceuticals with unprecedented properties and therapeutic outcomes.

## 2. Methodology

**Figure 1:**
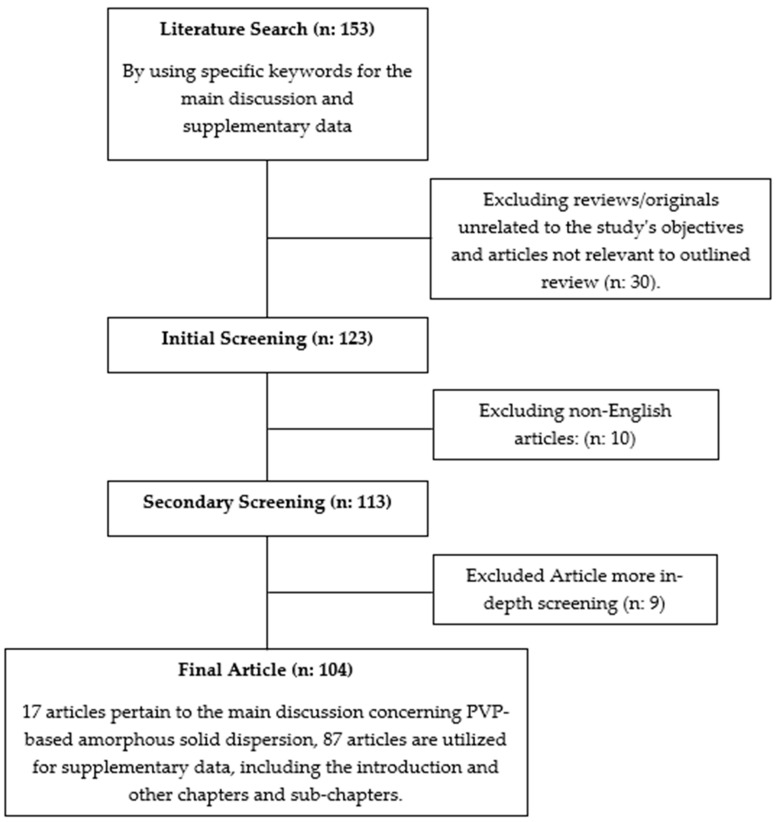
Flowchart of the methodology(“n” represents a variable. supplementary data represent all discussion from the introduction till the main discussion in this article).

The literature review conducted for this study involved searching for articles using the keywords “PVP Amorphous Solid Dispersion” in the Scopus, PubMed, and Google Scholar databases. Only relevant articles focusing on pharmaceutical formulations, specifically related to the physicochemical properties and strategies for enhancing the efficacy of active pharmaceutical ingredients, were included. Opinions, reviews, and unrelated topics were excluded. The flowchart of the methodology can be seen in Figure 1.

## 3. Current Techniques to Improve Drug Solubility for Drug Design and Development

### 3.1. Salt Modification

Salt modification is a common strategy used in the pharmaceutical industry to optimize the properties of drugs. It involves the conversion of a drug’s active pharmaceutical ingredient (API) into a salt form via reactions with an appropriate acid or base [26]. Salt modification of drugs has several key points worth noting. Firstly, the purpose of salt formation is to enhance the solubility, stability, and bioavailability of drugs. This alteration of a drug’s physicochemical properties leads to improved performance. Additionally, the salt form of a drug may exhibit pH-dependent properties, which can be advantageous for designing specific delivery systems such as delayed-release or targeted-release formulations. Another benefit is the stability improvement offered by salt formation, protecting drugs from degradation and extending their shelf-life, in addition to enhancing its bioavailability and increasing its absorption and therapeutic efficacy. When selecting the appropriate counterion for salt formation, factors like solubility, stability, taste, and toxicity are carefully considered. Some common salt forms include hydrochloride, sulfate, acetate, citrate, and maleate, named after the counterion used. Importantly, it is crucial to understand that the salt form of a drug does not affect its pharmacological activity, as the therapeutic effect primarily stems from the active moiety of the drug, irrespective of its salt form [27,28,29].

Salt modification is just one of the many techniques employed in pharmaceutical development to optimize drug properties and improve patient outcomes. Extensive studies, including preclinical and clinical evaluations, are conducted to ensure the safety and efficacy of salt-modified drugs before they are approved for use.

### 3.2. Structure Modification

Drug structure modification involves making deliberate and purposeful alterations to the chemical structure of a drug. It involves altering specific functional groups, substituents, or other molecular features to improve various properties, such as potency, selectivity, pharmacokinetics, or pharmacodynamics [30,31,32].

In drug structure modification, various approaches and techniques are commonly employed. One such approach is functional group modification, where altering or introducing functional groups in a drug molecule can impact its solubility, activity, metabolic stability, or target interaction [20]. Substituent variation involves replacing or modifying substituents attached to the drug’s core structure, influencing steric, electronic, or hydrophobic properties, which can affect target binding affinity or selectivity. Another technique is ring system modification, wherein changes to the size, aromaticity, or fusion of rings can impact the drug’s conformation, receptor binding, or metabolic stability [33]. Isosteric replacement involves substituting a specific atom or functional group with an isosteric counterpart, maintaining molecular shape and size while altering other properties to optimize metabolic stability, reduce toxicity, or enhance target binding [34]. Prodrug design involves modifying the drug structure to create inactive or less active forms that undergo conversion in the body, thereby improving drug delivery, solubility, and bioavailability, and enabling tissue targeting or bypassing metabolic barriers [35]. Lastly, conformational modification plays a crucial role, as altering the three-dimensional shape of a drug molecule through structural changes or introducing constraints can enhance binding affinity, selectivity, and stability during interaction with target proteins [36]. It is important to note that drug structure modification is a complex and iterative process that often involves a combination of synthetic chemistry, computational modeling, and biological evaluation [37]. It requires extensive research, including structure–activity relationship (SAR) studies and in vitro/in vivo testing, to understand the impact of structural changes on drug properties and optimize the desired characteristics [38,39].

### 3.3. Particle Size Reduction

Drug particle size reduction is a technique used to decrease the size of drug particles to achieve certain desired properties or improve drug performance. It involves reducing the particle size of the active pharmaceutical ingredient (API) to increase its surface area, enhance dissolution rate, improve bioavailability, or enable formulation into various dosage forms [40]. Particle size reduction of drugs is aimed at enhancing dissolution and absorption characteristics [41]. Smaller particle size increases surface area, improving solubility, dissolution rate, and absorption speed. Various techniques are utilized for particle size reduction, including mechanical milling, high-pressure homogenization, micronization, and nanosizing. The impact of particle size reduction on dissolution rate and bioavailability is significant, especially for drugs with low solubility. However, formulation considerations must be taken into account, such as stabilization, control of particle size distribution, and selection of suitable excipients to prevent aggregation and ensure uniformity in dosage forms [42]. Challenges include optimizing the process to achieve desired particle size distribution, preventing agglomeration or attrition, and maintaining chemical and physical stability [43]. Regulatory authorities may require characterization and documentation of particle size distribution and its effect on drug performance, particularly for generic drug products seeking approval [44,45].

It is important to note that particle size reduction should be carefully evaluated in terms of its impact on drug stability, formulation feasibility, and other relevant factors [46]. The specific technique chosen for particle size reduction depends on the properties of the drug, the desired particle size range, and the intended application.

### 3.4. Nanoparticle Drug Delivery System

Nanoparticle drug delivery systems refer to the use of nanoparticles as carriers or vehicles for delivering drugs to specific target sites in the body. These systems utilize nanoscale particles, typically ranging from 1 to 100 nanometers in size, to encapsulate, protect, and deliver therapeutic agents. Nanoparticle drug delivery systems offer numerous advantages. Their small size provides a high surface area-to-volume ratio, facilitating high drug loading and protection against degradation. Nanoparticles can be functionalized to enable targeted drug delivery, binding specifically to desired cells or tissues, reducing off-target effects and systemic toxicity. Liposomes, polymeric nanoparticles, lipid nanoparticles, dendrimers, carbon nanotubes, and inorganic nanoparticles are common types used in drug delivery, each with tailored properties. Encapsulation of drugs within nanoparticles enhances stability, controls release kinetics, and improves solubility, especially for hydrophobic drugs. Controlled and sustained drug release can be achieved by manipulating nanoparticle composition, size, surface properties, and structure. Nanoparticles can also possess imaging or diagnostic capabilities, allowing simultaneous drug delivery and real-time monitoring of therapeutic response. Challenges include scalability, reproducibility, biocompatibility, and regulatory approval, in addition to the needs for safety and toxicity assessments for long-term effects on the body [47,48,49,50].

Nanoparticle drug delivery systems represent an active area of research and development in the field of pharmaceutical sciences. They hold great promise for improving the efficacy and safety of therapeutic interventions by enabling targeted and controlled drug delivery.

### 3.5. Co-Crystallization

Drug co-crystallization is a technique used to improve the physicochemical properties of drugs by forming crystalline complexes with other molecules called co-formers. Co-crystals are solid-state structures composed of the drug molecule and the co-former, held together by non-covalent interactions such as hydrogen bonding, π-π stacking, or Van der Waals forces.

Co-crystals can modify melting point, dissolution rate, crystal habit, polymorphism, and solid-state stability. Co-formers, which are small organic molecules, interact with drugs through non-covalent interactions, and to do so, they depend on their ability to form stable co-crystals and their compatibility with the drug. Co-crystals are formed by mixing drugs and co-formers in a suitable solvent or through grinding, crystallization, or co-evaporation. Co-crystallization impacts drug properties, enhances solubility and stability, modulates polymorphism, and improves bioavailability. Characterization techniques like X-ray crystallography, differential scanning calorimetry (DSC), X-ray diffraction (XRD), Fourier-transform infrared spectroscopy (FTIR) and solid-state nuclear magnetic resonance (NMR) can be used to confirm the presence of co-crystals and their structure, while in vitro and in vivo evaluations assess the co-crystallization effect on drug performance. It is interesting to note that co-crystal formation may generate new intellectual property rights, as the composition and properties of the co-crystal can be patented to protect the innovation [9,51,52].

Co-crystallization is a versatile tool in drug development and can be applied to a wide range of drug molecules. It offers opportunities for optimizing drug properties, overcoming formulation challenges, and improving therapeutic outcomes.

### 3.6. Solid Dispersion

Drug solid dispersion refers to a formulation technique to enhance the solubility, dissolution rate, and subsequently the bioavailability of poorly water-soluble drugs by dispersing them in a solid matrix. In a solid dispersion, drugs are dispersed at the molecular or microscale level within a carrier matrix to increase their surface area for dissolution. The carrier matrix can be a hydrophilic or hydrophobic polymer or a combination of both, or lipid-based carriers for lipid-based solid dispersion. Solid dispersions can be prepared through melting, solvent evaporation, hot-melt extrusion, or spray drying methods. The dispersion enhances solubility and dissolution rate, leading to improved drug absorption and bioavailability. The stability of the drug within the solid dispersion is crucial and depends on the carrier, preparation method, and storage conditions. Formulation considerations include selecting the appropriate carrier, optimizing the drug-to-carrier ratio, and considering factors like compatibility, processing parameters, drug loading, and particle size distribution. Techniques such as DSC, XRD, FTIR, and microscopy are used to characterize solid dispersions, assessing the physical state and drug distribution within the carrier matrix [53,54,55,56].

Drug solid dispersion is a widely used technique in the formulation of poorly water-soluble drugs. It can overcome solubility limitations, enhance drug release, and improve therapeutic outcomes by increasing drug solubility and bioavailability.

## 4. Amorphous Solid Dispersion System

A formulation strategy known as amorphous solid dispersion (ASD) shows promise in improving the solubility, dissolution rate, and bioavailability of poorly water-soluble pharmaceuticals. However, due to the complexity of ASD’s physicochemical properties, resulting from different production methods and formulations, efficient characterization methods are needed to assess their physical stability. While ASD has been used to enhance the bioavailability of poorly water-soluble drugs, its physical instability currently limits its commercial use. In ASD systems, the conversion of the crystalline form to an amorphous state reduces the free energy, making ASD thermodynamically unstable compared to its crystalline counterpart, which has lower molecular energy and stronger molecular bonds.

Polymers are known as a suitable matrix-forming excipient in ASD and can prevent recrystallization of amorphous pharmaceuticals by increasing the viscosity below the T_g_ or interfering with polymer–drug interactions. Ideally, ASDs should remain kinetically stable during their predicted shelf-life, although they may slowly return to their crystalline state. The presence of water (such as high humidity) increases the mobility of ASDs and reduces the polymer’s ability to inhibit recrystallization [57].

The dissolution process of polymer–drug molecules revealed that crystalline drug structures with strong intramolecular hydrogen bonds would have lower solubility. To address limited water solubility in crystalline compounds, the solid dispersion approach combines crystalline active ingredients with amorphous polymers that are highly water-soluble. Molecular or non-molecular mixing of the active ingredient and polymer allows for certain potentially stable and strong interactions, particularly through hydrogen bonding.

The characterization of a drug–polymer complex in the development of solid dispersion showcases the molecular dispersion of the active ingredient within the polymer phase, while maintaining the main structure of the active pharmaceutical ingredients without any alteration, as observed through amorphous patterns in crystallography and differential scanning calorimetry. The molecular state of the active ingredient plays a key role in improving solubility, as it dissolves along with the polymer when exposed to the solvent medium.

Based on the characterized molecular state, the dissolution mechanism for amorphous solid dispersion was proposed as follows. Strong drug–polymer interactions in the solid state reduce phase separation in the presence of water and will enable a controlled drug dissolution by the polymer. Upon dispersion in the dissolution medium, the drug is rapidly released due to monomolecular dispersion, and the drug–polymer interaction maintains high drug supersaturation in the dissolution medium, resulting in a significant amount of dissolved drug in the medium. In contrast, the crystalline state of the drug exhibits lower equilibrium solubility; thus, a lower amount of drug is being released into the dissolution medium [58,59].

In conclusion, in the ASD system, the drug is dispersed in a polymer matrix in its amorphous form, meaning it lacks a well-defined crystal structure. By converting the drug into its amorphous form and dispersing it within a polymer matrix, the surface area available for dissolution is maximized, leading to an enhanced solubility and dissolution kinetics [17].

The polymer matrix in an amorphous solid dispersion serves multiple functions. It stabilizes the amorphous drug, preventing its recrystallization into a less soluble form. Additionally, the polymer can act as a solubilizing agent, aiding in drug dispersion and maintaining a high concentration gradient for dissolution. Commonly used polymers in ASD include hydrophilic polymers such as polyvinylpyrrolidone (PVP), hydroxypropyl methylcellulose (HPMC), polyethylene glycol (PEG), and copolymers like Soluplus^®^ and Eudragit^®^. In addition to choosing a suitable polymeric matrix, various other strategies can also be employed to maintain the stability of ASDs, including, among others, incorporating stabilizers or anti-recrystallization agents [59], optimizing storage conditions (e.g., moisture protection), or employing techniques like spray drying with rapid drying rates to minimize drug–polymer interaction time [7,8,60,61].

## 5. The Ideal Characteristics of Polymer and Drug for ASD

The successful design of an amorphous solid dispersion (ASD) hinges on the interplay of ideal characteristics within both the polymer and the drug. In terms of the polymer, optimal solubility and affinity are paramount, facilitating the formation of a molecular dispersion with the drug, ensuring a homogeneous distribution. Additionally, the polymer must possess the ability to maintain its amorphous state, preventing crystallization of both the polymer and the drug. A crucial parameter is the glass transition temperature (Tg), which should exceed the intended storage and processing temperatures to sustain the amorphous nature of the components [7].

Compatibility with manufacturing techniques is another key consideration; the polymer should align with common methods such as hot-melt extrusion or spray drying. Biocompatibility and safety of the polymer are imperative for adherence to regulatory standards and ensuring patient well-being [16].

Conversely, for the drug, characteristics such as low water solubility are advantageous as one of the primary goals of ASDs is to enhance solubility. A high melting point is beneficial for processes involving elevated temperatures, like hot-melt extrusion. Thermodynamic stability in the amorphous state, limited hygroscopicity to avoid stability issues, and compatibility with the polymer for stable molecular dispersion are vital drug attributes [62].

Furthermore, an ideal drug for ASDs should demonstrate a substantial improvement in bioavailability, often attributed to increased dissolution rates in its amorphous state. It is also critical that the drug maintains therapeutic efficacy within the polymer matrix, ensuring the desired therapeutic effect upon administration. Balancing these characteristics is pivotal for the successful development of ASDs, offering a platform for improved drug delivery and bioavailability in pharmaceutical formulations [62].

## 6. Preparation of Amorphous Solid Dispersion

The preparation of ASD involves several techniques that aim to convert the drug into its amorphous form and disperse it within a suitable polymeric matrix. Here are some commonly used methods for preparing ASDs:

### 6.1. Solvent Evaporation/Co-Solvent Method

The solvent evaporation/co-solvent method involves dissolution of drug and polymer(s) in a solvent or a mixture of solvents. The solvent is then evaporated, leaving behind a solid dispersion where the drug is dispersed in an amorphous form within the polymer matrix. Among the important steps in this method include selecting suitable solvent(s), dissolving the drug and polymer(s), evaporating the solvent, and collecting the resulting solid dispersion for further characterization and evaluation [58,63,64].

### 6.2. Melt Quenching Method

In the melt quenching method, the drug and polymer(s) are heated above their melting points to form a molten mixture. The molten mixture is then rapidly cooled or “quenched” to room temperature or below, which solidifies the mixture into an amorphous solid dispersion. This method allows for the conversion of the drug into its amorphous form and its dispersion within the polymer matrix. The resulting amorphous solid dispersion can be further characterized and evaluated for its physical properties and drug-polymer interactions [65,66].

### 6.3. Hot-Melt Extrusion (HME)

In this method, the drug and polymer(s) are fed into a co-rotating twin-screw extruder. The extruder applies heat and mechanical shear to melt the drug and polymer(s) and thoroughly mix them. The molten mixture is then forced through a die and rapidly cooled to solidify it into ASD. The resulting amorphous solid dispersion can be further characterized and evaluated for its physical properties and drug-polymer interactions [23,67,68].

### 6.4. Spray Drying

In this method, a solution or suspension of the drug and polymer(s) is atomized into small droplets using an atomizer or spray nozzle. These droplets are then exposed to a stream of hot air or inert gas in a drying chamber. As the droplets travel through the chamber, the solvent rapidly evaporates, leaving behind solid particles of the ASD. The resulting amorphous solid dispersion can be further characterized and evaluated for its physical properties and drug–polymer interactions [69,70,71].

It is important to note that the selection of the appropriate method depends on various factors, including the physicochemical properties of the drug and polymer(s), drug–polymer compatibility, desired dosage form and formulation characteristics, and processing capabilities. It is hence recommended to conduct feasibility studies and optimization experiments to determine the most suitable preparation method for a given drug and polymer combination [69,70,71].

During the preparation of ASD, it is crucial to maintain suitable processing conditions to prevent drug recrystallization and maintain the amorphous state. Characterization techniques such as DSC, XRD, FTIR, and microscopy are employed to confirm the amorphous nature of the drug and assess the physical state and distribution within the polymer matrix. Additionally, stability considerations, such as protection from moisture, light, and temperature, should be taken into account during storage and handling to ensure the stability of the ASD.

## 7. Characterization and Evaluation of Amorphous Solid Dispersion

Characterization and evaluation of ASDs are essential to assess their physical properties, drug–polymer interactions, and potential benefits in improving drug solubility, dissolution, and bioavailability. Here are some commonly employed methods for the characterization and evaluation of ASD.

### 7.1. Differential Scanning Calorimetry (DSC)

DSC is used to determine the thermal behavior of ASDs. It can identify changes in melting point (T_m_) and glass transition temperature (T_g_), and detect drug–polymer interactions, crystallization behavior, and stability of an ASD. DSC measures the difference in heat flow between a sample and a reference material as a function of temperature. When applied to ASDs, DSC can provide insights into the amorphous state of the drug and its interactions with the polymer matrix [15,72,73,74].

### 7.2. X-ray Diffraction (XRD) and Powder X-ray Diffraction (PXRD)

X-ray diffraction (XRD) analysis plays a crucial role in determining the crystallinity of a drug and evaluating the amorphous nature of a solid dispersion. ASDs typically exhibit a lack of distinct crystalline peaks compared to the crystalline drug. When applied to ASDs, XRD offers insights into the presence of a crystalline drug or polymer, the degree of amorphousness, and the physical stability of the formulation. It is essential to acknowledge the limitations of XRD, especially in detecting low levels of crystallinity or characterizing highly amorphous systems. In such instances, complementary techniques like solid-state nuclear magnetic resonance (SSNMR) spectroscopy or differential scanning calorimetry (DSC) can provide additional insights [15,74].

Powder X-ray diffraction (PXRD), on the other hand, serves as a valuable tool for determining the crystallinity of the drug and assessing potential drug recrystallization within the ASD. Despite the lack of long-range order in ASDs, PXRD can still offer valuable information regarding the presence of crystalline drugs or polymers, detect changes in crystallinity, and evaluate the physical stability of the formulation [74,75,76,77].

### 7.3. Fourier-Transform Infrared Spectroscopy (FTIR)

FTIR is a valuable tool for characterizing amorphous solid dispersions and providing information about the chemical composition, drug–polymer interactions, amorphousness, and stability of the formulation. It aids in understanding the molecular properties of ASDs and supports the development and optimization of drug delivery systems [15,74,78].

### 7.4. Scanning Electron Microscopy (SEM) and Transmission Electron Microscopy (TEM)

SEM and TEM provide morphological information about the solid dispersion, such as particle size, shape, crystallographic properties, and distribution of ASDs. They can reveal the presence of drug particles within the polymer matrix. Both SEM and TEM provide high-resolution imaging capabilities, but they differ in terms of sample preparation and imaging methods. Both SEM and TEM play complementary roles in characterizing amorphous solid dispersions. SEM provides valuable information about surface morphology and overall particle characteristics, while TEM allows for a more detailed investigation of the internal structure and nanoscale features. These techniques together contribute to a comprehensive understanding of the physical properties and stability of amorphous solid dispersions, which is crucial for the development of effective pharmaceutical formulations [15,74,79,80].

### 7.5. Solid-State Nuclear Magnetic Resonance (NMR)

Solid-state NMR spectroscopy can provide insights into drug–polymer interactions and molecular dynamics within the ASD. It can be used to study drug dispersion, drug–polymer interactions, and drug mobility, in addition to enabling in-depth analysis of the solid-state properties of ASD [15,74,81].

### 7.6. Dissolution Testing

Dissolution studies are crucial for evaluating the drug release behavior and solubility enhancement of ASD as compared to crystalline drugs. These studies provide information about the release profile, bioavailability, and effectiveness of the ASD formulation. The dissolution test measures the rate and extent of drug release from ASD in simulated gastric or intestinal fluids. It helps to assess the drug’s solubility, the ability of the ASD to improve dissolution, and the impact of formulation factors. The test generates dissolution profiles that allow for formulation optimization and understanding of drug release kinetics. Parameters such as dissolution rate, dissolution efficiency, and in vitro–in vivo correlation (IVIVC) can be determined, aiding in evaluating ASD performance and predicting pharmacokinetics. The dissolution test is an essential tool for developing and ensuring the quality of ASDs, optimizing drug solubility, and improving therapeutic outcomes [74,77,82].

## 8. Polyvinylpyrrolidone (PVP)

Polyvinylpyrrolidone (PVP) is a synthetic polymer characterized by its unique chemical structure, composed of repeating units of N-vinyl-2-pyrrolidone (NVP). Represented as (C₆H₉NO)n, where n denotes the number of repeating units, PVP exhibits a range of molecular weights, spanning from several thousand to over a million Daltons [83].

This polymer is known for its excellent solubility in water and various solvents, making it highly versatile in a multitude of applications across different industries. In the pharmaceutical sector, PVP serves as a binder in tablet and granule formulations, a disintegrant for promoting tablet disintegration, and a solubilizer to enhance the solubility of poorly soluble drugs [84].

Beyond pharmaceuticals, PVP finds applications in cosmetics, acting as a film-former in hair sprays and gels, and as a stabilizer in cosmetic formulations. In the food industry, it serves as a clarifying agent in beverages and as a stabilizer to enhance the texture of certain food products. Industrially, PVP is used as an adhesive component and in the textile industry for sizing and finishing processes [85].

Biocompatible and widely accepted by regulatory authorities, PVP undergoes polymerization, typically through free-radical processes, to yield its useful properties. The safety and efficacy of PVP have been extensively studied, contributing to its approval for use in various applications [86].

In summary, polyvinylpyrrolidone’s solubility, film-forming characteristics, and biocompatibility render it an invaluable polymer in pharmaceuticals, cosmetics, food, and industrial processes. Tailoring its molecular weight and grade allows for customization to meet specific requirements in diverse applications.

Polyvinylpyrrolidone (PVP) plays a significant role in the formulation of amorphous solid dispersions (ASDs), particularly in the pharmaceutical industry [87]. ASDs are designed to enhance the solubility and bioavailability of poorly water-soluble drugs, and PVP offers unique advantages in achieving these goals. As a carrier polymer, PVP’s high solubility in water enables the formation of molecular dispersions with hydrophobic drugs, helping to maintain them in an amorphous state [88]. The presence of PVP in ASDs acts as a stabilizer, preventing the crystallization of drugs and contributing to improved physical stability [89]. This stabilization is vital, as the amorphous form of a drug often exhibits higher solubility and dissolution rates, translating to enhanced bioavailability. PVP also facilitates ease of processing during manufacturing, compatible with techniques like spray drying and hot-melt extrusion, commonly employed for ASD production. The versatility of PVP extends to its compatibility with a wide range of drug molecules. Additionally, its biocompatibility ensures the safety of its use in pharmaceutical formulations. In summary, PVP’s solubilization properties, ability to stabilize the amorphous state, enhancement of dissolution rates, ease of processing, compatibility with diverse drugs, and biocompatibility make it a valuable component in the development of effective amorphous solid dispersions for improved drug delivery [90]. For ASD application in pharmaceuticals, many studies have reported the ability of PVP to produce ASD for different drug molecules, as summarized in Table 1.

## 9. Discussion

There are numerous studies that have reported on the application of PVP to produce ASDs for different drugs (Table 1). Different preparation methods were explored including solvent evaporation, melt quenching, electrospinning, and coprecipitation. Danda et al. (2019) explore the combination of different ASDs formulated with water-soluble and water-insoluble polymers to achieve and sustain supersaturation of the poorly soluble drug posaconazole (PCZ). ASDs of PCZ were prepared using PVP/VA64 or an ammonio-methacrylate copolymer (Eudragit^®^), and physical mixtures of these ASDs were also prepared. The ASDs were characterized using FTIR and PXRD, and their dissolution behavior was compared to that of crystalline PCZ. The results showed that both ASDs were in the amorphous state, and no crystalline PCZ was detected in the ASDs based on FT-IR analysis. The equilibrium solubility of crystalline PCZ varied depending on the pH, while all ASDs achieved higher concentrations than the equilibrium solubility of crystalline PCZ during dissolution. The PVP/VA64 ASDs exhibited better dissolution and slower recrystallization rates compared to the ASD prepared with Eudragit^®^ RS PO. Combining 20 mg PVP/VA64 with 80 mg Eudragit^®^ RS PO as PCZ carriers resulted in the highest area under the curve (AUC), indicating sustained supersaturation over time even after the complete dissolution of the PVP/VA64 component. The study demonstrates the potential of combining multiple ASDs to achieve specific dissolution profiles and enhance the solubility of poorly soluble drugs [58].

Another study was conducted by Wang et al. (2017), in which they used density functional theory (DFT) to predict polymer–drug interactions and investigated the ability of PVP to inhibit crystallization in amorphous solid dispersions. Solid dispersions of PVP/resveratrol (RES) and PVP/griseofulvin (GRI) were studied. DFT calculations indicated a strong interaction between PVP and RES, while a weaker interaction was observed between PVP and GRI. Experimental analysis using FTIR confirmed the hydrogen bonding between PVP and RES. Modulated differential scanning calorimetry (mDSC) and XRD showed 70–90 wt% PVP/RES and PVP/GRI dispersions formed amorphous solid dispersions. Under accelerated testing conditions, PVP/RES dispersions with higher miscibility (90/10 wt%) were more stable compared to PVP/GRI dispersions. The dissolution rate of PVP/RES dispersions remained high even after 90 days of storage due to the strong interaction between PVP and RES. In contrast, the dissolution rate of PVP/GRI dispersions significantly decreased due to GRI recrystallization [90].

Anon et al. investigated the clinical significance of reducing drug crystallinity in a furosemide–PVP (K-25) model solid dispersion system. X-ray amorphous and semi-crystalline dispersions of furosemide were compared to crystalline furosemide and an oral furosemide solution. The trial involved seven healthy male Caucasians, and each participant received different formulations on a single-blind basis. Urine samples were collected and analyzed for sodium and potassium levels to determine furosemide’s efficacy. The results indicated that the bioavailability of all formulations was equal and equivalent to an oral solution, suggesting a similar extent of absorption [54].

Weuts et al. [91] aimed to investigate the influence of intermolecular forces on the stability of loperamide and its fragment molecules (F1 and F2) in solid dispersions with PVP-K30 and PVP-VA64 in different storage conditions. Chemical stability was evaluated using HPLC, while TGA analysis measured water content and MT-DSC measurements assessed changes in the physical state of the compounds during storage. Results showed that dispersions with PVP-K30 absorbed more water in humid conditions compared to those with PVP-VA64, indicating the hydrophilic nature of the former polymer. This water acts as a plasticizing agent, increasing mobility and reducing the T_g_. The degree of supersaturation and molecular mobility were found to impact the stability of the amorphous state. The higher molecular mobility of F1 in the dispersions compared to F2 led to crystallization in F1/polymer dispersions stored under high relative humidity (52%). The formation of hydrogen bonds between F2 and the polymers reduced its mobility and prevented crystallization, highlighting the significance of specific interactions for stability in solid dispersions. On the other hand, F1, which did not form hydrogen bonds with the polymers, had compromised amorphous state stability and underwent crystallization. Loperamide, which also did not form hydrogen bonds, was less prone to crystallization due to its inherent glass-forming properties [91].

Ekdahl et al. focused on using the spray-drying process to engineer the properties of ASD containing felodipine and polyvinyl pyrrolidone vinyl acetate (PVP-VA). By adjusting the atomization and drying conditions, four different powders with varying particle properties were obtained. The particles exhibited a wide range of sizes, bulk densities, and morphologies. The powders showed different flowability characteristics, ranging from poor to easy flowing, but all samples were found to be suitable for producing strong tablets at reasonable compression pressures. The mechanical property tests indicated favorable tablet bonding and high brittleness for all powders. The study demonstrates that spray-dried ASD particles can be tailored to achieve desired powder flow and mechanical properties, thereby reducing processing risks and increasing process efficiency [92].

A study by Qian et al. [93] aimed to understand the unexpected differences in the bio-performance of two ASDs, BMS-A/PVP-VA and BMS-A/HPMC-AS, in dogs. The solubility of crystalline BMS-A in the polymers was measured, and drug–polymer interaction parameters were determined. Dissolution kinetics of the spray-dried dispersions were studied, and BMS-A supersaturation was assessed in the presence of pre-dissolved polymers. The potency and crystallinity of undissolved dispersions was also evaluated to determine the mechanism. The results showed that both polymers were miscible with BMS-A in the solid state, with PVP-VA exhibiting better drug solubility. In vitro dissolution of BMS-A was similar to both solid dispersions, but the HPMC-AS dispersion performed better in vivo. HPMC-AS was more effective in prolonging BMS-A supersaturation, yet its slow dissolution rate prevented BMS-A recrystallization within the matrix. On the other hand, fast dissolution of PVP-VA led to drug recrystallization and lower bioavailability. The study highlights the importance of polymer selection in solid dispersion development, as it affects both physical stability and in vivo performance [93]. In this case, PVP-VA is a less suitable choice as compared to HPMC-AS.

Lu et al. [94] investigated the mechanisms of sodium dodecyl sulfate (SDS) and PVP/VA in enhancing the dissolution of high-loaded felodipine (FLDP) amorphous extrudates. The rheological analysis shows that PVP/VA inhibits FLDP crystallization, and binary and ternary extrudates of FLDP-PVP/VA-SDS are ASDs. Internal SDS decreases the T_g_ of FLDP-PVP/VA ternary ASDs. The enhanced dissolution rate of PVP/VA-rich ASDs in the presence of SDS is observed. SDS enhances dissolution by improving wettability and forming complexes with PVP/VA, accelerating medium uptake and erosion kinetics. However, SDS can induce FLDP recrystallization and result in incomplete dissolution of FLDP-rich extrudates. Interestingly, a specific ratio of FLDP-SDS promotes drug dissolution. Tablet formulations with internally or externally added SDS confirm the in vitro relevance of these molecular interaction mechanisms, with internally added SDS significantly promoting FLDP dissolution [94].

Different from the other studies, Lafountaines et al. (2016) compared two thermal processing methods, hot-melt extrusion (HME) and KinetiSol^®^ Dispersing (KSD), for the production of ASDs containing griseofulvin (GRI). The influence of polymer type, polymer molecular weight, and drug loading on the dispersions’ properties was investigated. HME and KSD were used with PVP K17 or HPMC E5. HME produced amorphous dispersions at lower drug loads but showed crystallinity at higher drug loads. KSD produced amorphous dispersions at higher drug loads with higher-molecular-weight polymers that were not processable by HME. Various analytical techniques supported these findings [23].

Lehmkemper et al. (2017) studied the preparation and long-term physical stability of ASD formulations, with the aim to enhance the bioavailability of active pharmaceutical ingredients (APIs). The solubility of acetaminophen and naproxen in the polymer excipients PVP K25 and PVP-VA64 was calculated using three models: Perturbed-Chain Statistical Associating Fluid Theory (PC-SAFT), Flory–Huggins (FH) model, and an empirical model. PC-SAFT and FH were also used to predict the influence of relative humidity (RH) on API solubility in the polymers. The Gordon–Taylor equation was applied to estimate the T_g_ of dry ASDs under different humidity conditions. The calculations were validated through 18-month stability studies at standardized storage conditions. The results showed that the modeling approaches aligned with experimental solubility data, with FH predicting lower solubility at room temperature compared to PC-SAFT and the empirical model. RH was found to have a minor impact on acetaminophen solubility but was predicted to decrease naproxen solubility in the polymers, as confirmed by long-term stability studies. PC-SAFT demonstrated better agreement with the stability study results than FH [95].

Another study by Knopp et al. [22] aimed to investigate the impact of the copolymer composition of polyvinylpyrrolidone vinyl acetate (PVP-VA) on the dissolution behavior and in vivo performance of celecoxib (CCX) ASD. The results showed that the hydrophilic monomer vinylpyrrolidone (VP) contributed to the generation of CCX supersaturation, while the hydrophobic monomer vinyl acetate (VA) stabilized the supersaturated solution. The optimal copolymer composition was found to be around 50–60% VP content, beyond which further substitution of VP with VA did not provide any biopharmaceutical advantages. The study demonstrated a linear relationship between in vitro and in vivo performance, indicating that the non-sink in vitro dissolution method employed could predict the drug’s behavior in the body. These findings highlight the significant influence of copolymer composition on the dissolution profile and in vivo performance of amorphous solid dispersions, suggesting the possibility of tailoring drug dissolution by adjusting the monomer ratio in the copolymer to achieve the desired plasma concentration profile. Determining the optimal ratio for a specific drug is crucial, as it is likely to be drug-dependent [102].

The study related to this modification was performed by Knopp et al. (2016). They investigated that the impact of polyvinylpyrrolidone (PVP) molecular weight on the dissolution behavior and performance of celecoxib (CCX): PVP ASDs. The results show that as the molecular weight of PVP decreases, the dissolution rate of CCX increases, and the inhibition of crystallization is enhanced with the increasing molecular weight of PVP, reaching a maximum for PVP K30. This suggests that the relationship between crystallization inhibition and molecular weight is not linear, and there is an optimal molecular weight for the strongest inhibition. In line with the dissolution findings, ASDs with higher-molecular-weight PVP (K30 and K60) exhibit significantly higher in vivo bioavailability (AUC_0–24h_) compared to pure amorphous and crystalline CCX. The study establishes a linear correlation between in vitro and in vivo AUC_0–24h_, indicating that the non-sink in vitro dissolution method can effectively predict the in vivo performance of CCX: PVP ASDs, enabling formulation ranking. Overall, the molecular weight of polymers can significantly influence the in vitro and in vivo performance of CCX: PVP ASDs [22].

Yuan et al. reported a study that exploited the SSNMR technique to assess the miscibility of an ASD formulation prepared from nifedipine (NIF) and PVP through different methods. The compositions prepared by melt quenching in the lab setting showed that the 95:5 and 90:10 NIF: PVP dispersions were not miscible, while the 75:25, 60:40, and 50:50 compositions were found to be miscible based on 1HT1ρ relaxation measurements. The domain size of the miscible systems was estimated to be less than 4.5 nm. The 90:10 NIF: PVP dispersions prepared by spray drying and melt quenching in the NMR rotor also exhibited miscibility. Additionally, variable-temperature SSNMR measurements indicated a change in relaxation time below the T_g_, suggesting increased molecular mobility above that temperature [66].

Ball milling is a conventional method that is still applicable as a technology in preparing ASDs. A study by Caron et al. investigated the physicochemical properties of binary amorphous dispersions of poorly soluble sulfonamides with polymeric excipients prepared by ball milling. The sulfonamides tested included sulfathiazole (STZ), sulfadimidine (SDM), sulfamerazine (SMZ), and sulfadiazine (SDZ), using PVP and Soluplus^®^ (polyvinyl caprolactam-polyvinyl acetate-polyethylene glycol graft copolymer) as the polymer matrix. PXRD and DSC analysis confirmed the PVP ability to form amorphous dispersions over a wider composition range compared to Soluplus^®^, and this was reported for all sulfonamides tested in this study. The amorphous dispersions made with PVP were homogeneous with a single T_g_, while those made with Soluplus^®^ were heterogeneous with two T_g_ values, which could be attributed to the lower solubility of sulfonamides in Soluplus^®^ compared to PVP. Interestingly, even though SDM was not amorphized alone upon milling and Soluplus^®^ has a lower T_g_ than SDM, amorphous dispersions of SDM with Soluplus^®^ could still be produced. Amorphous dispersions of SMZ required a lower excipient concentration compared to STZ, SDM, and SDZ, possibly due to the one-dimensional H-bonding network in SMZ. Stability tests under high-humidity storage conditions (60%RH/25 °C) showed that dispersions made with Soluplus^®^ remained dry and powdery, while those made with PVP formed a sticky paste within 2 weeks, indicating better physical stability for Soluplus^®^ [97].

Bejaoui et al. (2022) also explored the ball milling technique in ASD preparation. They investigated the effect of adding PVP K30 to a binary solid dispersion of indomethacin (IND)/kaolin for the formation of a physically stable amorphous drug. The mixtures were ball-milled at room temperature, and the obtained materials were characterized using various techniques. The results showed that indomethacin interacts with kaolin and PVP K30 through hydrogen bonding, without any polymorphic transformations or chemical degradation. PVP acts as a linker between the drug and kaolin, enhancing the physical stability of the amorphous IND even under high-stress conditions. The ternary system (IND/kaolin/PVP) significantly improves drug solubility compared to the binary solid dispersion (IND/kaolin). The addition of PVP not only stabilizes the amorphous IND but also helps overcome solubility challenges associated with kaolin [98].

The impact of compression is also an important factor that needs to be evaluated in the formulation of ASDs. Ayenew et al. [99] investigated the miscibility of naproxen (NAP)-PVP K25 solid dispersions following compression. Different compositions of NAP and PVP K25 were subjected to compression at varying forces. The miscibility of the solid dispersions was evaluated using mDSC, and the specific interactions between NAP and PVP K25 were analyzed using FTIR. The results showed that the solid dispersion containing 20% (*w*/*w*) naproxen maintained a single T_g_ before and after compression, with the FTIR profile remaining unchanged. However, the compositions with 30% and 40% (*w*/*w*) naproxen exhibited significant changes in miscibility due to compression. Compression pressures beyond 565.05 MPa led to apparent amorphous–amorphous phase separation, characterized by two distinct Tg values in the mDSC and altered FTIR spectral profile. The flexible nature of PVP facilitated plastic deformation during compression, resulting from the rotation of the PVP backbone and changes in dihedral angles. This plastic deformation, including segmental rotation, could increase the structural temperature and potentially affect the miscibility by weakening or disrupting intermolecular hydrogen bonding between the drug and polymer upon compression [103].

Electrospinning is a relatively newer method in ASD. It started to gain popularity in 2021 when Bitay et al. [100] first reported their success in producing ASD with this method. Since then, many studies have explored the potential of electrospinning for ASD with various drugs and polymers. Study has focused on the preparation of lapatinib-loaded nanofibrous solid dispersions using PVP through the electrospinning technique. The aim was to enhance the aqueous solubility and dissolution rate of the anticancer drug. The nanofibers exhibited smooth surfaces with homogeneous filaments, with an average diameter of 462 ± 160 nm, as determined by SEM. DSC confirmed the transition of the active ingredient from crystalline to amorphous form, and Raman spectroscopy revealed a uniform distribution of amorphous lapatinib within the fibrous structures. Gas chromatographic analysis showed that residual solvents in the nanofiber mats were below recommended limits. The lapatinib content in the nanofibers was measured at 16.76 ± 0.11% (*w*/*w*), and in vitro dissolution studies demonstrated a rapid release of approximately 70% of the drug within 5 min at pH 6.8, compared to the minimal dissolution of pure lapatinib ditosylate. These findings highlight the potential of electrospinning in improving the physicochemical characteristics of poorly bioavailable anticancer agents [100].

A study by Martínez-Ohárriz et al. [101] investigated the effectiveness of the coprecipitation technique in the preparation of ASD from diflunisal and PVP K15, K30, and K90, as well as physical mixtures. The materials were analyzed using XRD, FTIR, DSC, and hot-stage microscopy. The XRD results showed that even coprecipitates with a high drug content (70%) were almost amorphous, regardless of the polymer molecular weight. FTIR spectra indicated the formation of diflunisal–PVP hydrogen bonds in the 70:30 drug–polymer solid dispersions. The dissolution kinetics of the physical mixture with a 70:30 drug–polymer ratio was linear, while the corresponding coprecipitates exhibited two different dissolution processes. Hot-stage microscopy of the 25:75 drug–polymer dispersion showed only solid plates of PVP, suggesting a molecular dispersion of the drug in the polymer. Polymorphic changes in diflunisal were observed in the solid dispersions compared to the physical mixtures, which were consistently composed of polymorph II. At high drug concentrations (75:25 and 80:20), the XRD patterns of the solid dispersions indicated partial recrystallization of the drug. Ethanol and chloroform as coprecipitation solvents resulted in different diflunisal polymorphs (polymorph I and form IV, respectively) [101].

## 10. Authors’ Perspective

The aim of this review is to explore and investigate the application of ASD in improving the dissolution and bioavailability of poorly soluble drugs, focusing on PVP or its derivatives (e.g., PVP-VA, PVP-K series) as the polymer matrix. The polymer matrix is important for facilitating the formation of an amorphous state and maintaining drug stability.

**Figure 2:**
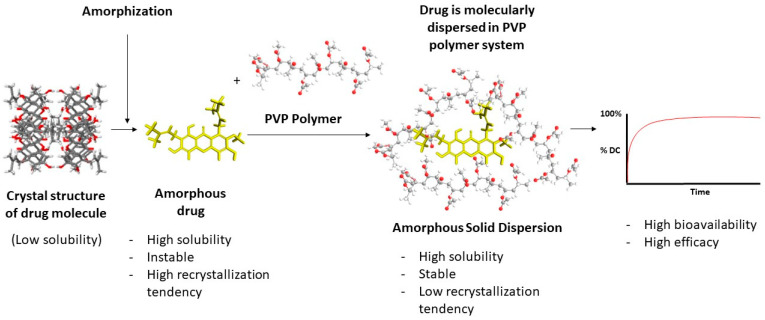
Amorphous solid dispersion.

Based on the available literature, ASD systems have demonstrated improved physicochemical properties and effectiveness of the tested drug as compared to their crystalline form. They achieved higher drug concentrations during dissolution, sustained supersaturation, enhanced solubility, and increased dissolution rates, which is due to their ability to prevent or slow down drug recrystallization and maintain supersaturation, leading to improved bioavailability. The specific combination of polymers and drug carriers in the ASDs contributed to these characteristics. The illustration of an ASD system can be seen in Figure 2.

The mechanism behind the improvement of physicochemical properties and effectiveness by ASD systems involves various factors. PVP-based ASDs inhibit drug crystallization and thus maintain the drug in an amorphous state. They enhance drug dissolution by improving wettability, forming hydrogen bonds or specific interactions with the drug, increasing drug solubility, and promoting drug–carrier complexation.

Many studies share the objective of utilizing ASDs to enhance drug dissolution and bioavailability, which can be achieved using polymers such as PVP-based polymers. The potential of ASD in pharmaceutical applications, as presented in this review, has indeed provided valuable insights into the design and development of ASD systems for poorly soluble drugs. The adoption of ASD to optimize drug performance has received validation, as evidenced by the successful progression of several products through clinical trials and their subsequent successful introduction to the pharmaceutical market. Notable examples include Kaletra^®^ for HIV/AIDS, where ASD has proven effective in improving the solubility and bioavailability of lopinavir. Similarly, Reyataz^®^ for HIV/AIDS has demonstrated enhanced solubility and bioavailability of atazanavir, and Prezista^®^ for HIV/AIDS has exhibited heightened bioavailability of darunavir. Moreover, Vfend^®^, employed as an antifungal agent, has showcased improved solubility and absorption of voriconazole through ASD. Immunomodulatory drugs such as Gengraf^®^, Neoral^®^, and Sandimmune^®^ have utilized ASD to augment the solubility and bioavailability of cyclosporine. The application of ASD in Invirase^®^ for HIV/AIDS has also proven instrumental in enhancing the solubility and absorption of saquinavir. These instances underscore the current efficacy of ASD, positioning it as a promising and impactful technique for ongoing and future development of pharmaceuticals.

## 11. Conclusions

In conclusion, ASD is an effective technique to improve the dissolution and bioavailability of poorly soluble drugs, and PVP-based polymers have been shown to play a crucial role in the formation of ASD through maintaining the amorphous state of the drug and inhibiting drug recrystallization. ASDs can provide enhanced dissolution rates, sustained supersaturation, increased drug concentrations during dissolution, and subsequently improved bioavailability, which can be attributed to factors like improved wettability, specific interactions between drug and polymer, and inhibition of drug recrystallization. These findings highlight the potential of ASDs as a strategy to enhance the effectiveness of poorly soluble drugs in vivo.

## Figures and Tables

**Table 1:** The development of PVP-based ASDs.

No	Active Substance	Structure	Method	Study Objective	Result	Reference
1.	Posaconazole (PCZ)	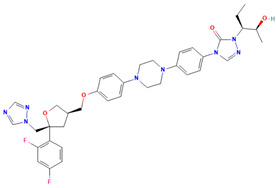	Solvent evaporation	Explored combinations of water-soluble and water-insoluble polymers in ASDs for PCZ.$$$Used PVP/VA64 and ammonio-methacrylate copolymer (Eudragit^®^).	Demonstrated enhanced dissolution and sustained supersaturation, with improved stability using a combination of PVP/VA64 and Eudragit^®^ RS PO.	[58]
2.	Resveratrol (RES) and Griseofulvin (GRI)	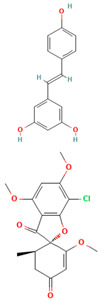	Solvent evaporation	Utilized density functional theory (DFT) to predict polymer–drug interactions.$$$Studied PVP/RES and PVP/GRI ASDs.	Highlighted the impact of polymer–drug interactions on stability and dissolution rates.	[90]
3.	Furosemide (FUR)	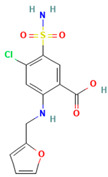	Solvent evaporation	Investigated the clinical significance of reducing drug crystallinity in PVP-FUR model solid dispersions.	Conducted a trial with different formulations and found bioavailability to be equal, suggesting similar absorption characteristics.	[54]
4.	Loperamide, and (4-dimethylamino-*N*,*N*-dimethyl-2,2-diphenyl-butyramide and 4-(4-chlorophenyl)-4-piperidinol	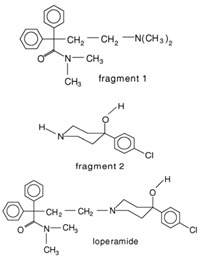	Spray drying	Explored the influence of intermolecular forces on loperamide stability in solid dispersions with PVP-K30 and PVP-VA64.	Highlighted the impact of hydrogen bonds on stability and crystallization in different storage conditions.	[91]
5.	Felodipine	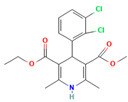	Spray drying	Used the spray-drying process to engineer ASDs containing felodipine and PVP-VA.	Demonstrated tailoring of particle properties for desired powder flow and mechanical properties, indicating potential for efficient tablet production.	[92]
6.	BMS-A fromBristol-Myers Squibb	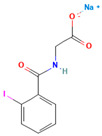	Spray drying	Explored the unexpected differences in the bio-performance of two ASDs: BMS-A/PVP-VA and BMS-A/HPMC-AS.	Highlighted the importance of polymer selection in solid dispersion development for physical stability and in vivo performance.	[93]
7.	Felodipine	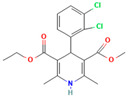	Hot-melt extrusion	Investigated the mechanisms of sodium dodecyl sulfate (SDS) and PVP/VA in enhancing the dissolution of high-loaded felodipine amorphous extrudates.	Demonstrated enhanced dissolution rates through improved wettability and molecular interactions.	[94]
8.	Griseofulvin	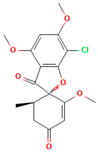	Hot-melt extrusion and KinetiSol^®^ Dispersing	Compared hot-melt extrusion (HME) and KinetiSol^®^ Dispersing (KSD) for ASDs containing griseofulvin.	Investigated the influence of polymer type, molecular weight, and drug loading on dispersion properties.	[23]
9.	Acetaminophen and$$$naproxen	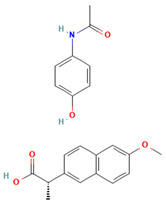	Hot-melt extrusion$$$	Studied the preparation and long-term physical stability of ASD formulations containing acetaminophen and naproxen in PVP K25 and PVP-VA64.	Validated modeling approaches through stability studies.	[95]
10.	Celecoxib	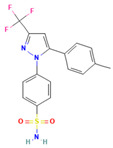	Melt quenching	Investigated the impact of PVP-VA copolymer composition on the dissolution behavior and in vivo performance of celecoxib ASD.	Demonstrated the influence of copolymer composition on dissolution profiles and in vivo performance.	[96]
11.	Celecoxib	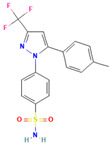	Melt quenching	Explored the impact of PVP molecular weight on the dissolution behavior and performance of celecoxib: PVP ASDs.	Established a correlation between molecular weight and in vitro/in vivo performance.	[22]
12.	Nifedipine	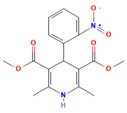	Melt quenching, spray drying, and melt quenching, spinning	Explored the miscibility of an ASD formulation prepared from nifedipine and PVP using solid-state NMR.	Indicated miscibility based on relaxation measurements and domain size estimation.	[66]
13.	Sulfonamide	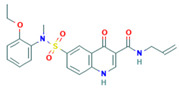	Ball Milling	Investigated the physicochemical properties of sulfonamide–PVP ASDs prepared by ball milling.	Compared PVP and Soluplus^®^ as polymeric excipients and highlighted better physical stability for Soluplus^®^.	[97]
14.	Indomethacin (IND) and Kaolin	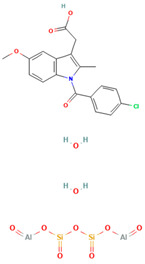	Ball milling	Explored the effect of adding PVP K30 to a binary solid dispersion of IND/kaolin for the formation of a physically stable amorphous drug.	Demonstrated enhanced drug solubility and stability through hydrogen bonding.	[98]
15.	Naproxen	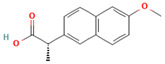	Compression	Investigated the miscibility of naproxen (NAP)-PVP K25 solid dispersions following compression.	Highlighted the impact of compression on miscibility and the potential role of plastic deformation in altering drug–polymer interactions.	[99]
16.	Lapatinib	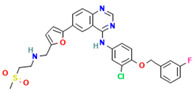	Electrospinning setup	Explored electrospinning for the preparation of lapatinib-loaded nanofibrous solid dispersions using PVP.	Demonstrated improved physicochemical characteristics and dissolution rates for poorly bioavailable anticancer agents.	[100]
17.	Diflunisal	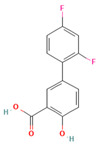	Coprecipitation	Investigated the effectiveness of coprecipitation technique in the preparation of ASD from diflunisal and PVP K15, K30, and K90.	Highlighted the impact of polymer molecular weight on amorphous dispersion properties.	[101]

## Data Availability

Not applicable.
